# Insights from the national maternal and Child Health Workforce Development Center on Title V Teams’ collaborative readiness and goal accomplishment

**DOI:** 10.1007/s10995-022-03437-y

**Published:** 2022-04-27

**Authors:** Rebecca Wells, Alexandria M. Coffey, Amy Mullenix, Jessica Simon, Kristen Hassmiller Lich

**Affiliations:** 1 Department of Management, Policy, and Community Health, University of Texas School of Public Health, 1200 Pressler Street, 77030 Houston, USA; 2 Department of Maternal and Child Health, University of North Carolina – Chapel Hill, 135 Dauer Drive, 27599 Chapel Hill, North Carolina USA; 3 The National MCH Workforce Development Center, University of North Carolina – Chapel Hill, 135 Dauer Drive, 27599 Chapel Hill, North Carolina USA; 4 The Association of Maternal and Child Health Programs, 1825 K Street Suite 250, 20006-1202 Washington, DC United States; 5 Department of Health Policy and Management, University of North Carolina – Chapel Hill, McGavran-Greenberg Hall, CB# 7411, 27599-7411 Chapel Hill, NC USA

**Keywords:** Maternal and child health, Partnerships, Collaboration, Readiness, Workforce development

## Abstract

**Purpose:**

State Title V programs collaborate with diverse partners to improve maternal and child health. Since 2014, the National Maternal and Child Health Workforce Development Center has trained Title V leaders in facilitating system change. This article describes aspects of initial collaborative readiness differentiating state and jurisdiction teams that later reported meeting their goals to greater or lesser degrees.

**Description:**

We used quantitative data from initial team leader reports to characterize readiness to collaborate with external partners, and their responses twelve months later to a prompt about how fully they had accomplished their goals. In addition, we coded excerpts from team leader accounts six and twelve months into their work with the Center, and retrospective coach perspectives, to identify collaborative readiness patterns.

**Assessment:**

Teams whose leaders reported higher goal accomplishment twelve months after beginning work with the Center had initially reported higher levels of collaboration with key partners. Our analyses suggest that such teams were also better able to use their cohort experience with the Center to improve collaboration, including information sharing with external stakeholders. Challenges working with Medicaid were reported both by teams with more and less goal accomplishment.

**Conclusions:**

Title V teams with lower levels of initial collaborative readiness may benefit from additional support in skill development, connections to key partners, and convening power. Given the crucial and increasing role of Medicaid in maternal and child health systems, more attention may be warranted to supporting all Title V programs in partnering with this funder.

## Significance

**What is already known on this subject?:**

Prior research has identified the ability to convene diverse stakeholders as key to achieving partnership synergies, and in turn improved community outcomes.

**What this study adds:**

State and territorial Title V programs may achieve greater synergies with external partners by initially assessing and strategically enhancing collaborative readiness. Training and technical assistance providers might enhance partnership synergies through focused assistance to states with lower initial levels of external collaboration. Title V leaders may improve outcomes by leveraging their strongest collaborations to foster relationships with additional stakeholders. Title V programs in general could benefit from support cultivating relationships with Medicaid.

## Introduction

The Health Resources and Services Administration’s Maternal and Child Health Bureau (HRSA MCHB) administers the Title V MCH Block Grant program as a state-federal partnership focused on the health of mothers, children, and families (HRSA MCHB 2018). A central function of Title V is convening across systems. In order to address determinants of health effectively, Title V professionals often seek to collaborate with counterparts in other areas of health and human services.

Defining collaboration as partnerships in which human and material resources are combined to accomplish objectives partners could not achieve alone, Lasker, Weiss, and Miller (2001, p. 183) describe “partnership synergy” as the “extent to which the perspectives, resources, and skills of its participating individuals and organizations contribute to and strengthen the work of the group.” Based on prior literature, they conclude that synergy is the mechanism through which partnership functioning (*what partners do*) becomes partnership effectiveness (*what they achieve*) and describe a framework of determinants of partnership synergy that has been supported by more recent evidence (Butterfoss, Goodman, & Wandersman, 1996; Fawcett et al., 1997; Gray, 1989; Roussos & Fawcett, 2000; Silka, 1999).

In addition to tangible resources including funding and other supports (Allen, Javdani, Lehrner, & Walden, 2012; Flewelling & Hanley, 2016; Kramer et al., 2005; Marchand, Fowler, & Kokanovic, 2005; Stevens, Rice, & Cousineau, 2007; Teaster & Wangmo, 2010), key determinants of partnership synergy include connections to other organizations (Allen et al., 2012; Flewelling & Hanley, 2016; Valentijn et al., 2015), ability to convene diverse stakeholders (Kramer et al., 2005; Marchand et al., 2005; Stevens et al., 2007), leaders’ skills and expertise (Allen et al., 2012; Feinberg, Greenberg, & Osgood, 2004; Marchand et al., 2005; Powell & Peterson, 2014; Stevens et al., 2007), internal organization and structure (Chutuape et al., 2015; Flewelling & Hanley, 2016; Valentijn et al., 2015), and information development (Allen et al., 2012). Resulting partnership synergies can include thinking about issues holistically, incorporating diverse stakeholder perspectives, and developing common goals (Lasker, Weiss, & Miller, 2001). These studies document impacts across a variety of dimensions of goal achievement, including partnership strengthening, partner benefit, enhanced action, and improved health outcomes.

Training 45 Title V teams in leading intersectoral initiatives since 2014 uniquely positions the National Maternal and Child Health Workforce Development Center to learn how collaborative readiness may lead to later goal accomplishment. Intensive training cohorts are the key mechanism used by the Workforce Development Center to enhance the existing MCH workforce’s skills in three core areas: systems integration, change management/adaptive leadership, and evidence-based decision making. This model responds to needs in the MCH field for advanced skills that can support upstream work, achieve equity, and impact outcomes. The 8-month intensive cohort experience includes cross-sector MCH state teams who convene to work on a health transformation challenge of their choice. The challenge serves as a “learning lab” for the skills taught in the three core areas. The cohort experience begins with two months of pre-work to help teams clarify their goals and team composition. Throughout this experience teams work in partnership with an assigned team coach who brokers resources and supports the team. Early on, the teams participate in an in-person learning institute, with didactic and applied learning opportunities for teams to apply the content to their challenge. The learning institute is followed by other learning modalities such as webinars and in-state consultations.

Our collaborative readiness training includes assessing the “5Rs” in the systems teams seek to improve (desired **R**esults; participant **R**oles; available **R**esources; **R**elationships among participants; and **R**ules shaping roles, resources, and rules); mapping networks of ties among team members; using causal loop diagrams to depict feedback loops; practicing conversational capacity for mutual learning; and activating partnerships.

In this article, we highlight initial collaborative readiness differences between 19 state and jurisdiction teams with more versus less subsequent goal accomplishment from 2015 to 2017 intensive training cohort evaluation results.

## Methods

After omitting one state that did not achieve its goals during their first project year and one state whose team leaders could not respond at the 12-month interview, our sample comprised of 19 state and jurisdiction teams participating in intensive Center cohorts between 2015 and 2017. The 19 states and jurisdictions represented all regions of the United States (3 northeastern, 8 southern; 4 mid-western; 4 western). The percentages of their populations living in rural areas ranged from 5 to 62% (less than 10%: 3, 10-19.9%: 3, 20-29.9%: 5, greater than 30%: 8). Of these, we had leader responses about collaboration with partners that coaches identified as key to their particular projects for eight teams, which we examined more closely (Yin, 2017). These teams’ goals included developing toolkits for breastfeeding and telehealth implementation for children and youth with special health care needs (CYSHCN) and increasing state collaboration for serving children in low income families and CYSHCN.

Data about partnerships are collected prior to, during, and after the cohort experience. Data from all three time points were used in these analyses. Surveys completed prior to the cohort experience assess the status of each team’s partnerships and ask teams to provide an example of a collaborative and challenging partnership. Teams reports on the status of these partnerships near the end of the 8-month intensive cohort experience and complete telephone interviews approximately six months after that experience, during which their original partnership responses are reviewed and discussed. The researchers used interview transcripts to examine differences in how partnerships were described.

We used two types of Center evaluation data for all 19 states and jurisdictions. First, we averaged team responses on a 1–5 scale (1: we do not collaborate, 2: not very collaborative, 3: neutral, 4: collaborative, 5: very collaborative) characterizing their levels of collaboration with key partners, such as Medicaid/State Children’s Health Insurance Program (SCHIP), private payers, federally qualified health centers, and professional organizations. These data were extracted from a survey completed collaboratively by each team as they were beginning intensive work with the Center. Second, we used team leader responses from a phone interview 12 months later to a prompt about whether they had achieved their goals ‘to a very high degree’ or ‘somewhat’ to indicate level of goal accomplishment. The response options were: ‘beyond what we could have imagined achieving’ (n = 0); ‘to a very high degree’ (n = 10); ‘we somewhat reached out goals’ (n = 9); ‘we did not reach our project goals’ (n = 1, excluded from analysis); and ‘can’t answer at this time, but confident we will reach our project goals’ (n = 1, excluded from analysis). We compared states and territories reporting goal accomplishment ‘to a very high degree’ to those who reported having ‘somewhat’ accomplished their goals.

For the eight states and jurisdictions examined more closely, we also used qualitative data from team presentations made to Center staff and the rest of their cohorts six months after project initiation, describing accomplishments, tools, knowledge, and skills gained throughout the intensive Center cohort, and in phone interviews between team leaders and Center staff 12 months after project initiation. The intensive cohort experience entailed four days of on-site training, with a common curriculum for all teams, followed by tailored coaching as each team began the project they had chosen to develop their leadership capacity, along with monthly webinars for all teams.

We coded the qualitative data in two ways. First, based on the team presentations six months after project initiation, we constructed binary indicators of six elements of progress we identified as related to collaboration: (1) building relationships and convening stakeholders; (2) engaging key players using Center tools; (3) using system integration tools taught by the Center; (4) understanding issues around health transformation; (5) progress in strategic thinking; and (6) understanding needs for solving MCH challenges. Second, we used Lasker, Weiss, and Miller’s determinants and elements of partnership synergy (2001) as a framework for coding the six-month team presentations and structured interviews with Center evaluation staff 12 months after project initiation and reflections elicited from coaches in preparation for this paper. Interview guides were shared with team leaders in advance to help solicit feedback from all team members. Questions were developed based on the primary goals and objectives of the Center, including how the Center increased their workforce development capacity, influenced health transformation, strengthened partnerships, and advanced their MCH population health goals. We dropped, added, and revised codes through individual review and discussion, to fit the current data (Miles, Huberman, & Saldaña, 2014). The Office of Human Research Ethics at the University of North Carolina at Chapel Hill reviewed the Center’s evaluation, as the manuscript is not based upon clinical study or patient data, and determined it was exempt from IRB approval.

## Results

### Higher goal accomplishment linked to initial levels of collaboration and use of training to improve collaboration and information sharing

As predicted by Lasker, Weiss, and Miller (2001), the current evaluation indicated that Title V connections to other organizations as they began work with the Center affected their subsequent goal accomplishment. Initial team leader ratings of partnerships averaged 3.7 on a 1 (‘we do not collaborate’) – 5 (‘very collaborative’) scale among teams whose leaders later reported achieving their goals to ‘a very high degree,’ slightly higher than the 3.5 mean among teams with later reports of ‘somewhat’ reaching goals (Table 1). For the eight teams for whom the partners we had asked all teams about were identified by coaches as key to their projects, the difference was greater, at 3.5 for those with a high subsequent degree of goal accomplishment versus 3.0 for those somewhat reaching their goals.

**Table 1:** Level of collaboration at project initiation by goal accomplishment status, 2015–2017

	Mean	Standard deviation
All state and jurisdiction teams (n = 19)	3.6	0.6
A very high degree (n = 10)	3.7	0.4
Somewhat (n = 9)	3.5	0.8
Selected sample (n = 8)	3.3	0.7
A very high degree (n = 4)	3.5	0.4
Somewhat (n = 4)	3.0	0.9

In keeping with the differences in initial ratings of levels of collaboration, the higher goal achievement teams wrote in an average of 2.8 names of additional partners beyond our standard list, whereas those later only ‘somewhat’ achieving their goals wrote in an average of 0.5 additional partner. This difference appeared to reflect more convening power among the high goal achievement teams (Lasker et al., 2001). In turn, work with the Center could enhance convening power: “For our MCO’s [managed care organizations], for our other partners, for those that we were seeking partnership with that we had an experience in the past. I think, really, the Center gave us that credibility that helped to bring them to the table.”

Some of the teams that later somewhat achieved their goals also reported gaining convening power through their work with the Center. As one such team leader put it, “There were some other tools that [a coauthor of this article] introduced to us altogether with partners from across government and nonprofit organizations, and it not only just helped all of us to work together, but it also gave a really good impression to our director.” The leader of another state that somewhat achieved its goals described using work with the Center to bring a major partner back to the table who had lost interest in the work, and recommitting.

Two states illustrate initial collaborative readiness leading to more successful convening of external partners, and ultimately higher reported goal attainment. One state initially reported collaborative or very collaborative relationships with key partners, including several they added to the list provided. They noted that the stakeholder meeting they held during a site visit by Center trainers “really solidified [the agency] as a key player in the early childhood health system.” Information sharing facilitated by the Center included “conversations with the leads with a lot of these programs … that also helped to illuminate everybody’s role in this…” This team later reported having achieved their goals ‘to a very high degree.’ In contrast, another state initially characterized only one partnership as collaborative, and did not list any additional partners. They engaged multiple sectors throughout their work with the Center, but framed cooperation in terms of aspirations rather than progress (“I think these efforts will really lend themselves towards a little bit better cooperation”). They later reported somewhat reaching their goals.

An exception to high goal achieving teams’ generally positive reports of initial partnerships was Medicaid, although the one initiative with an explicit Medicaid focus began with a collaborative relationship with this funder and ended with high reported goal accomplishment. Four of the eight teams we examined more intensively– two out of four of both with those with higher and lower later goal accomplishment – reported challenges working with Medicaid. No other type of partner was identified as challenging by more than one state and jurisdiction. As the leaders of one of the teams later reporting high goal accomplishment noted, “One of our primary clients is children and families enrolled in Medicaid and SCHIP. In [state] the [Medicaid agency] develops policies and collects data on this population. This information and input from [Medicaid agency] staff is valuable to the work we do. However, much of the time they are short staffed and do not readily identify with our public health goals.” A leader in another team noted challenges related to frequent Medicaid policy changes.

In general, teams with higher goal achievement were more likely than those with lower goal accomplishment to report building relationships and convening stakeholders (3 of the 4 among those with the highest goal accomplishment vs. 2 of the 4 among those who somewhat achieved their goals) and engaging key players using Center tools (all 4 among those with high goal accomplishment, vs. 2 of the 4 who somewhat achieved their goals) (Table 2). However, there was no difference between the two goal accomplishment categories of teams in their reported use of systems integration tools or understanding of issues related to health transformation, and the teams with lower goal accomplishment actually more frequently reported gains in two other more general aspects of strategic thinking.

**Table 2:** Team presentation findings 6-months after project initiation by goal accomplishment status, 2015–2017

	A very high degree (n = 4)		Somewhat (n = 4)	
	**Frequency**	**Percentage**	**Frequency**	**Percentage**
Built relationships and convened stakeholders	3	75%	2	50%
Enhanced ability to engage key players using Center tools	4	100%	2	50%
Used systems integration tools	2	50%	2	50%
Enhanced understanding of issues around health transformation	1	25%	1	25%
Enhanced strategic thinking	1	25%	2	50%
Increased understanding of needs for solving MCH challenges	0	0%	1	25%

Team leaders also reported leveraging their work with the Center to improve collaboration, a dynamic in which the states and jurisdictions with higher later goal accomplishment appeared to be more successful during their intensive cohort experience. In turn, this could lead to seeing maternal and child systems more holistically and identifying common goals. As the leader of a team with high goal accomplishment observed, “I think the project allowed us to bring the leadership across all health and education together, look at how we can align our programs and our needs to serving our families in a more systematic way. And I think as a result of that, it gave us skills across our programs to even look at how we are leaders within our organization, but also as we serve the bigger early childhood system.” Similarly, the leader of a state that later somewhat achieved its goals described clinicians and public health practitioners seeing issues “from different perspectives” through a meeting convened with the Center. However, they seemed to be at a different stage in the collaborative process. Among their insights six months after beginning work with the Center: “If we can’t engage an entire system, start somewhere and build momentum.”

We found more qualitative evidence that teams ultimately reporting high goal accomplishment used their work with the Center for sharing information with partners. As one high goal accomplishment leader observed about her team, “…they’re more comfortable working together and supporting and participating and providing more information in their initiative.”

A leader in another state reflected that “And going through that sustainability assessment tool, we really came to recognize the fact that we needed to just expand our involvement of community members beyond those that were already someway involved with children that have special healthcare needs, that we felt like there was a definite need to increase that awareness through marketing and use of media to communicate that value out of it to those folks in the community.”

The coach of a team with high goal accomplishment reported that “…sharing information about their transformation process … was DIRECT info elicitation/sharing, facilitated by a center tool.” In contrast, the coach of a team that later reported somewhat achieving their goals observed that “this team did not share Center tools and skills with partners outside of their team,” although “these skills and tools are still influencing work beyond Title V through the work of individual team members.” The coach of another team that later reported somewhat achieving their goals did report ongoing communication with clinic partners.

Overall, it appears that initial levels of collaborative readiness position Title V teams to make greater gains in partnership synergy, leading to higher levels of goal accomplishment. Teams in both goal accomplishment categories, however, reported challenges working with Medicaid.

## Discussion

Through our analysis of evaluation results from 19 state and jurisdiction teams participating in intensive cohorts with the National Maternal and Child Health Workforce Development Center between 2015 and 2017, we discovered higher initial collaboration and more subsequent information sharing and additional relationship building among teams with higher self-rated goal accomplishment 12 months after project initiation. Teams with high goal accomplishment named more partners at the outset of their project in a structured assessment and anecdotally reported more initial credibility with partners than teams who somewhat achieved their goals. Teams with high goal accomplishment were also more likely to report having learned about how to build relationships, convene stakeholders, and engage key players using Center tools. In essence, initial collaborative readiness appears to affect success in relationship-building and convening efforts. As Title V programs initiate new projects, it may be beneficial to begin by assessing and improving collaborative readiness. Teams with lower initial collaborative readiness may benefit from additional support in developing leadership skills, connecting to external stakeholders, and achieving the convening power to engage them.

We also discovered that teams with high goal accomplishment were more likely than teams who somewhat achieved their goals to leverage their work with the Center to improve collaboration, share information with partners, and increase stakeholder engagement in their initiatives. Center tools for eliciting diverse perspectives on maternal and child health systems appeared to be useful in part because they provided external validation of the state and jurisdictional Title V programs. These were generally useful to all teams, but more so for those with higher initial collaborative readiness.

One opportunity to support Title V teams across levels of collaborative readiness is in their partnerships with Medicaid, as this was identified as challenging even by some teams later reporting high goal accomplishment. Lasker, Weiss, and Miller (2001) discussed relationships among partners partly in terms of terms of power differentials and respect. Medicaid budgets dwarf those of Title V, creating a major power differential. Anecdotally, there is not so much evidence of distrust between Medicaid and Title V, as a lack of recognition of public health in general, and Title V specifically, related to Medicaid’s mission to provide ‘medically necessary’ care for individuals. This may be an increasing communication challenge as Title V continues to shift toward population health improvement. Such lack of recognition in turn has implications for Title V community support, as limited partnerships may undermine policy comprehensiveness (Lasker et al., 2001). In order to cultivate effective partnerships with Medicaid, Title V leaders may need more information about Title V effects on health care costs, skills in making the case for the medical necessity of holistic systems, and connections to brokers who can advocate on their behalf. The Center and other technical assistance providers assist Title V in addressing these challenges by enhancing convening power and encouraging identification of mutual goals between Title V and Medicaid. Such skills and status may also help Title V leaders work more fully with schools, another key partner that is becoming more challenging to access as their administrators contend with increasingly vulnerable children and adversarial political climates.

One practical implication of this study is that cross-sector MCH initiatives should pay close attention to collaborative readiness prior to committing to work together, or early in the relationship. This might involve initial assessments of readiness or partner mapping prior to launching into the project work. These types of early collaboration activities can help build trust and make explicit the often assumed roles each partner will play in a collaborative effort.

This study was limited by the small sample and exclusive focus on states and jurisdictions engaged in an intensive cohort training and coaching experience with the National Maternal and Child Health Workforce Development Center. The small sample made tests of statistical significance between teams with more and less goal accomplishment infeasible. Generality to Title V programs that are not engaged in intensive training is unknown. The differential goal accomplishment documented among these states and territories may be in part a lifecycle issue. Some teams working with the Center have reported increasing impact after the one year mark, while initially successful initiatives may also lose momentum over time.

Collaboration within and among organizations is essential to addressing complex determinants of health. In this article, we have reported some preliminary patterns from our work with state and jurisdiction Title V teams building their skills in this type of leadership. We hope that, over time, such investigations will enable more public health leaders to translate partnership synergies into sustainable conditions for child and family health.

## Data Availability

Not applicable.
